# A novel anti-inflammatory natural product from *Sphaeranthus indicus* inhibits expression of VCAM1 and ICAM1, and slows atherosclerosis progression independent of lipid changes

**DOI:** 10.1186/s12986-015-0018-1

**Published:** 2015-06-05

**Authors:** Rai Ajit K. Srivastava, Sapna Mistry, Somesh Sharma

**Affiliations:** Department of Pharmacology, Piramal Life Sciences Ltd, Mumbai, India; Present address: Integrated Pharma Solutions, Philadelphia, Department of Pharmacology & Physiology, Drexel University School of Medicine, Philadelphia, USA; Present address: BioMarin Pharmaceuticals, Novato, CA USA

## Abstract

A large body of evidence suggests that atherosclerosis is an inflammatory disease, in which cytokines and growth factors play a major role in disease progression. The methanolic extracts of *Sphaeranthus indicus* as well as its active ingredient, 7-hydroxy frullanoide (7-HF), are shown to suppress LPS-induced cytokine production from mononuclear cells, and inhibit the expression of VCAM1, ICAM1 and E-selectin by TNF-α- stimulated HUVECs in a concentration-dependent manner. We tested the hypothesis that the inhibition of cytokines and adhesion molecules should attenuate the progression of atherosclerosis, independent of changes in the lipid profile. Studies were carried out in two animal models: a high fat-fed LDLr^-/-^ mouse and a high fat-fed hyperlipidemic hamster. Methanolic extract of *S. indicus* was dosed to hyperlipidemic LDLr^-/-^ at 100 and 300 mg (equivalent to 20 and 60 mg 7-HF)/kg body weight/ day for 8 weeks, and plasma lipids as well as aortic lesion area were quantitated. Hyperlipidemic hamsters were treated with one dose of 200 mg/kg/day. *S. indicus* extract treatment did not alter the lipid profile in both animal models, but reduced aortic lesion area in LDLr^-/-^ mice and hyperlipidemic hamsters by 22 % and 45 %, respectively. Fenofibrate, included as a reference agent, decreased aortic lesions by 26 % in LDLr ^-/-^ mice and 84 % in hyperlipidemic hamsters, respectively, which was driven by massive reductions in proatherogenic lipoproteins. The lipid-independent anti-atherosclerotic activity of *S. indicus* was associated with the reductions in the circulating levels of MCP-1, TNF-α, and IL-6 via phosphorylation and degradation of IkB-α that prevents translocation of NF-kB in the nucleus to induce proinflammatory cytokines. Our findings demonstrate that anti-inflammatory agents that lower pro-inflammatory proteins inhibit the progression of atherosclerosis. The methanolic extract of *S. inducus*, currently being used to treat psoriasis, offer promise to benefit individuals who have high circulating pro-inflammatory cytokines, and predisposed to coronary artery disease.

## Introduction

A number of studies for the past 15 years suggest that atherosclerosis, the main cause of coronary artery disease (CAD), is an inflammatory disease in which inflammation plays a key role in setting the stage as well as causing the progression of atherosclerosis (Reviewed in [1–4]. Immune cells are predominantly present in the early atherosclerotic lesions, and their effector molecules have been shown to accelerate progression of the lesions leading to acute coronary syndrome [3, 5]. Thus, immune mechanisms interact with metabolic risk factors to initiate, propagate, and activate lesions in the arteries. In addition to vascular endothelial and smooth muscle cells, blood borne inflammatory and immune cells constitute an important part of an atheroma, which is preceded by an accumulation of lipid-laden cells in the subendothelium [3, 6–9]. Endothelial cells recruit leukocytes by selectively expressing major adhesion molecule on the surface. Examples of specific adhesion molecules involved in initiation of atherosclerotic plaques include vascular cell adhesion molecule-1 (VCAM-1), intercellular adhesion molecule (ICAM1), and endothelial cell selectin (E-selectin) [8, 9]. The chemoattractant cytokine, monocyte chemoattractant protein-1 (MCP-1), interacts with the monocyte chemokine receptor CCR2, recruiting the monocytes to the arterial endothelium and facilitating their entry in the subendothelial space [9, 10].

During the past one decade, inflammatory nature of atherosclerosis has attracted basic, translational, and clinical researchers to find scientific basis leading to a robust link between inflammatory biomarkers and cardiovascular disease (CVD) in outcome studies. Towards this end, high sensitivity c-reactive protein (hsCRP), an acute phase reactant released during inflammatory processes [11, 12], has been recognized as a powerful predictor of traditional markers of cardiovascular risk [13–15]. Basic and clinical research data suggest that treatment with statins (3-hydroxy-3-methylglutaryl coenzyme A reductase inhibitors) to lower low-density lipoprotein (LDL) cholesterol levels also reduces hsCRP [16]. Additional support for the anti-inflammatory and immunimodulatory actions of statins came from clinical research. Thus, the magnitude of risk reduction associated with statin therapy may exceed that expected on the basis of the LDL-C lowering alone. Prospective evidence provided by the JUPITER trial (Justification for the Use of Statins in Primary Prevention: an Intervention Trial Evaluating Rosuvastatin) demonstrated that patients with normal LDL-C levels but elevated hsCRP levels showed highly significant (-44 %) reduction in adverse cardiovascular events [17], suggesting additional benefit of hsCRP reduction and demonstrating an inflammatory component in the CVD risk.

Immune-mediated inflammatory disease, including atherosclerosis, psoriatric arthritis (PsA) [18–21], and rheumatoid arthritis (RA) [22–26], are characterized by common inflammatory morbidity and mortality. Chronic activation of innate and adaptive inflammatory pathways that provide an essential defense against “foreign” substances ranging from bacterial products to endogenous oxidized lipids, may contribute to atherosclerotic plaque progression, destabilization, and ultimately rupture with subsequent clinical sequelae such as myocardial infarction or stroke [20, 27].

Recently, it was demonstrated that the extract of *Sphaeranthus indicus* as well as its active ingredient, 7-HF, a sesquiterpene lactone, inhibits the LPS-induced release/synthesis of several pro-inflammatory mediators such as TNF-α, IL-1β and IL-6 in freshly isolated human peripheral blood monocytes [28]. Moreover, both of these prevented constitutive proinflammatory cytokine production in primary cultures of rheumatoid synovial cells, and oral administration of 7-HF effectively suppressed the clinical signs of established arthritis in DBA/1 collagen-induced arthritis model [29]. However, the antiatherosclerotic activities of *S. indicus* extract and 7-HF have not been evaluated. We hypothesized that the anti-inflammatory activities of *S. indicus* extract and 7-HF, shown to cause lowering of VCAM1, ICAM1, and E-selectin, may inhibit the progression of arterial lesion formation. To test this hypothesis, we employed two widely studied animal models, LDLr ^-/-^ [30] and hyperlipidemic hamsters [31], and evaluated antiatherosclerotic activities of *S. indicus* extract. Our results show that the antiatherosclerotic efficacy of *S. indicus* methanolic extract occurs via attenuation of proinflammatory cytokines and adhesion molecules, and is independent of changes in the plasma lipid profiles.

## Materials and methods

### Reagents

RPMI 1640 medium, anti-β-actin, anti-histone, phenazine methosulfate (PMS), Dulbecco’s phosphate buffered saline (DPBS) and fetal bovine serum (FBS) were purchased from Sigma. Calpain Inhibitor I, and antibodies against IκBα phosphorylated IκBα p65 were obtained from Calbiochem (Merck Biosciences). Anti-ICAM-1 (clone BBIG-I1) anti-VCAM-1 (clone BBIG-V1) and anti-E-selectin (clone BBIG-E4), isotype control mouse IgG_1_ (clone 11711.11), the secondary antibody (anti-mouse IgG-HRP antibody) and bacteria derived recombinant human TNF-α were products of R&D Systems (Minneapolis, MN). Protease inhibitor cocktail was procured from Roche. The CellTiter 96^®^ Aqueous One Solution Cell Proliferation Assay were purchased from Promega (Madison, WI).

### Source of *Sphaeranthus Indicus* and 7-hydroxy frullanolide

Methanolic extract of the fruits of *Sphaeranthus Indicus* was prepared in-house and dissolved in DMSO as a 20 mg/ml stock as described [28]. 7-hydroxy frullanolide (7 HF), isolated in-house from the above plant was dissolved in DMSO as a 20 mM stock. It was purified and identified with the use of ESI-MS and ^1^H- and ^13^C-NMR analyses [28].

### Human peripheral blood mononuclear cells assay

Human peripheral blood mononuclear cells (PBMC) were harvested using Ficoll- Hypaque density gradient centrifugation (1.077 g/ml; Sigma Aldrich; St. Louis, MO) from healthy volunteers and suspended in assay medium [RPMI 1640 culture medium (Sigma Aldrich) containing 10 % heat inactivated fetal bovine serum (FBS; JRH Biosciences; Lenexa, KA), 100U/ml penicillin (Sigma Aldrich) and 100 μg/ml streptomycin (Sigma Aldrich)]. PBMC (2 × 10^5^) per well were transferred into a 96-well plate. The cells were pre-treated with varying concentrations of 7HF, *S. indicus* extract, or 0.5 % dimethyl sulfoxide (DMSO) or 10 μM 4- (4-fluorophenyl)-2-(4-methylsulfinylphenyl)-5-(4-pyridyl) imidazole [SB203580; a p38 MAPK inhibitor which is known to suppress induced production of TNF-α and IL-6; Sigma Aldrich] for 1 h at 37 °C, 5 % CO_2_ and stimulated with 1 μg/ml lipopolysaccharide (LPS; Escherichia coli serotype 0127:B8; Sigma Aldrich). The cells were incubated for 6 h at 37 °C, 5 % CO_2_ followed with collection of supernatants and assayed for TNF-α, IL-6, IL-8, and IL-1β by Enzyme-Linked Immunosorbent Assay (ELISA; OptiEIA ELISA sets; BD Biosciences). The 50 % inhibitory concentration (IC_50_) values were calculated by a nonlinear regression method using GraphPad software (Prism 3.03). In all experiments, a parallel plate was run to ascertain the toxicity of 7HF. The toxicity was determined using the CellTiter 96® AQ_ueous_ One Solution Cell Proliferation Assay (Promega; Madison, WI). In every experiment, each condition was run in triplicate wells.

### Endothelial cell culture

Human umbilical vein endothelial cells (HUVECs) and the complete medium were obtained from Cascade Biologics (Portland, Oregon). Cells were grown in endothelial cell growth medium M200 supplemented with 2 % low serum growth supplements as per the manufacturer’s recommended protocol. The growth medium was changed every other day until confluence. Cells under passage 8 were used for this study. The cells used for the experiments had a viability >98 % as determined by trypan blue exclusion test.

### Evaluation for the viability of endothelial cells

The CellTiter 96® Aqueous One Solution Cell Proliferation Assay (Promega) was used to assess cell viability. The assay is composed of the tetrazolium compound MTS (3-(4,5-dimethylthiazol-2-yl)-5-(3-carboxymethoxyphenyl)-2-(4-sulfophenyl)-2H-tetrazolium, inner salt) and an electron coupling reagent (PMS). The soluble product in the medium was measured with a spectrophotometer at 490-nm absorbance. Background absorbance from the control wells (same media, no cells) was subtracted. Cells incubated in control media were considered 100 % viable.

### Cell enzyme-linked immunosorbent assay

The surface expression of endothelial cell adhesion molecules was quantified using cell enzyme-linked immunosorbent assay. Briefly, confluent HUVECs in 96-well fibronectin–coated plates were pretreated with various concentrations of *S. indicus* or 7-HF for 30 min before being stimulated with 1 ng/mL TNF-α for the indicated time. The expressions of ICAM-1 and E-selectin were evaluated after TNF-α stimulation for 4 h, and expression of VCAM-1 was evaluated after 6 h of stimulation. The cells were fixed with 1 % paraformaldehyde and the non-specific binding of antibody was blocked using bovine serum albumin (2 % in DPBS). The cells were then washed and incubated with monoclonal mouse anti-human ICAM-1, VCAM-1, E-selectin or the isotype control mouse IgG1 overnight at 4 °C. Subsequently, cells were washed and incubated with a horseradish peroxidase-conjugated goat anti-mouse IgG for 90 min. Binding of the secondary antibody was determined by incubating with 3,3′,5,5′ tetramethylbenzidine (TMB) substrate from BD Biosciences (SD California) and then terminating the reaction by 2 N sulphuric acid. Surface expression of adhesion molecules was quantified by measuring absorbance at 490 nm in an automated microtitre plate reader (Spectramax, Molecular Devices, USA).

### IkBα phosphorylation

To assay IκBα, cytoplasmic extracts were prepared from cells pretreated with either *S. indicus* extract or 7 HF for 2 h and then stimulated with TNF-α either in the presence or absence of ALLN, a calpain inhibitor, resolved on 10 % sodium dodecyl sulfate-polyacrylamide gels. After electrophoresis, the proteins were transferred to nitrocellulose membrane, probed with antibodies against either IκBα or phosphorylated IκBα at serine 32, and detected by chemiluminescence (ECL; Sigma).

### NF-κB protein localization

For the determination of NF-κB localization, Western blot analysis was carried out with cytoplasmic and nuclear extracts using anti-human NF-κB primary antibody. These extracts were prepared as per manufacturers protocol (Chemicon). Briefly, treated cells were lysed in 300 μl of hypotonic lysis buffer containing 10 mM HEPES (pH 7.9), 1.5 mM MgCl_2_, 10 mM KCl, 0.5-5 mM DTT, 0.1 % Triton X-100, sodium orthovanadate, 1 mM and protease inhibitor cocktail. The residual pellet after cytosolic fraction collection was treated with extraction buffer containing 20 mM HEPES (pH7.9), 1.5 mM MgCl_2_, 0.42 M NaCl, 0.2 mM EDTA, 0.5-5 mM DTT, 1.0 % NP-40, 25 % (v/v) glycerol, sodium orthovanadate, 1 mM, and protease inhibitor cocktail. Twenty microgram protein were taken for Western Blot analysis as described above.

### In vivo cytokine production study

Group of 10 mice were treated orally with Sphaeranthus Indicus methanolic extract, 60 min prior to lipopolysaccharide injection (1 mg/kg, i.p.). Levels of TNF-α in the plasma, were measured 1.5 h after lipopolysacchride injection, and IL-1β were done 4 b after LPS treatment.

### Real-time quantitative PCR analysis

Quantitation of messenger RNA (mRNA) was done in aortic total RNA by real-time quantitative RT-PCR using an ABI Prism 7700 sequence detector. All PCR reactions were performed in a total volume of 50 μl and included the following components: cDNA derived from 20 ng of total RNA, 400 nM each of forward and reverse primers, RNase-free water, and 25 μl of Power SYBR Green PCR Master Mix (ABI), an optimized buffer system containing AmpliTaq Gold DNA polymerase and dNTPs. All PCR reactions were performed in quadruplicate using pooled cDNA samples (n = 6). Cycling parameters were as follows: after an initial denaturation step for 10 min at 95 °C, 40 subsequent cycles were performed in which samples were denatured for 15 s at 95 °C followed by primer annealing and elongation at 60 °C for 1 min. Relative quantities of mRNA were calculated from C_T_ values using the comparative C_T_ method (ΔΔC_T_; [32] using GAPDH as an internal reference. Primer pairs for real-time PCR were designed using Primer3 software and sequence information obtained from GenBank (NCBI).

### Atherosclerosis intervention study in hamsters

#### Animals and diet

All animal procedures were performed as per guidelines provided by the Institutional Animal Care and Use Committee. Golden Syrian (GS) hamsters (10-12 weeks old) obtained from Charles River, were housed in groups of three in appropriately sized solid-bottom cages with contact bedding. Room lighting conditions were adjusted to 12 h light and 12 h dark cycle as follows: light between the hours of 6 AM to 6 PM and dark between the hours of 6 PM to 6 AM. All animals were allowed to acclimate for 5 days in the vivarium and were fed standard rodent chow unless otherwise mentioned before initiating the study.

### Study details

After the acclimation period, baseline blood chemistry was done in animals after 12-h fasting followed by blood (700 μl – 800 μl) withdrawal by retro-orbital puncture in a 3 ml Vacutainer® tube containing K_3_EDTA. Blood samples were centrifuged (4000 rpm, 20 min, 4 °C) in an Eppendorf tube to obtain plasma, which was transferred (250–300 μl) into a clean tube. The aliquot of plasma sample was analyzed for triglycerides, total cholesterol, LDL-C, direct-HDL-C, glucose, AST and ALT. LDL, triglycerides, cholesterol, and glucose were quantitated on automated chemistry analyzer, Hitachi 917, using Roche diagnostic kits (Indianapolis). After bleeding, animals were earmarked for identification and then were returned to their cages and feed replaced. All animals were put on a high fat diet consisting of Purina 5001 plus 10 % coconut oil, 10 % corn oil, 0.5 % cholesterol, and 5 % fructose. This diet composition accelerates hyperlipidemia in hamsters. Animals were fed high fat diet for 4 weeks. This study started with a total of 50 animals. At the end of 4 weeks of feeding high fat high cholesterol diet, the hamsters were fasted overnight and bled as described above. Plasma samples were analyzed for total cholesterol, triglycerides, LDL-C, HDL-C, glucose, AST and ALT as described above. Based on the body weight, and blood chemistry those animals that did not develop adequate hyperlipidemia (total cholesterol >1000 mg/dl and total triglycerides >1200 mg/dl) were excluded from the study before grouping [33]. Animals were then randomly grouped into 3 groups of 10 animals in each group as follows: group 1- vehicle control CMC 0.5 %; group 2- fenofibrate 100 mg/kg/day; group 3- methanolic extract of *S. indicus* 200 mg/kg/day. Animals were fed high fat high cholesterol diet and concomitantly dosed by oral gavage once daily in the morning between 8 and 9 AM. At the end of 10 weeks of treatment, hamsters were euthanized under CO_2_ and blood withdrawn by cardiac puncture and processed as described above. The thorax was opened and vasculature perfused first with heparinized saline (40 units/ml) for 2 min and then with 10 % formalin for 5 min. The aorta attached to heart and containing aortic arch, thoracic and abdominal aorta to the femoral artery bifurcation were removed and placed in 10 % formalin for en face staining and lesion quantitation. Aortic lipid contents were quantitated by en face staining with Oil Red O and atherosclerotic lesion area coverage determined by image analysis.

Isolated aortae from the above study were placed on to a tray containing black wax. Connective tissues sticking around the artery were removed as much as possible. Abdominal aorta and renal arteries, iliac bifurcation, aortic arch and major branches were exposed. The aorta was snipped at the heart where it leaves the heart. Major branches were snipped at approximately 0.5 cm away from where it joins the aorta. Iliac arteries were snipped from the bifurcation point. The aorta was placed into 10 % formalin for lesion measurement.

Each section of the aorta was opened longitudinally and pinned to a black wax plate using stainless steel pins. The plate containing the aorta sections was then rinsed with 70 % ethanol (5 min), immersed in Oil Red O staining solution (10 min) followed by destaining with 70 % ethanol on a shaker for 5 min at room temperature. The aorta was rinsed with deionized water and then submerged with PBS. Quantitation and imaging was done as described [33]. Atherosclerosis burden was expressed as % lesion per area.

### Atherosclerosis intervention study in LDLr^-/-^ mice

#### Animals and diet

Male LDLr^-/-^ mice (C57Bl/6 J background) were procured from Jackson Laboratories (Bar Harbor, Maine) at 6 weeks of age. Mice were allowed to acclimatize on regular chow diet for one week followed by feeding a high fat high cholesterol (HF) diet with 45 % calories from butter fat plus 0.21 % cholesterol for 2 weeks to acclimatize to the high fat diet. On an average, the food consumption was around 3 g/day. One group of mice was fed rodent chow, Purina 5001 (*n* = 11).

### Study details

After the acclimation period of one week, baseline blood chemistry was done in animals after 4-h fasting followed by blood (150 μl) withdrawal by retro-orbital puncture tube containing K_3_EDTA. Each blood sample was centrifuged (4000 rpm, 20 min, 4 °C) using an Eppendorf tube, and the supernatant transferred into a new clean tube. The aliquot of plasma sample was analyzed for triglycerides and total cholesterol. After bleeding, animals were earmarked for identification and then were returned to their cages and feed replaced. All animals were put on a high fat diet consisting of Purina 5001 plus 21 % fat, and 0.21 % cholesterol.

The LDLr ^-/-^ mice on high fat diet were divided into 3 groups (*n* = 11/group) as follows: Group 1- Vehicle, high fat high cholesterol (HF) with 45 % calorie from butter milk and 0.21 % cholesterol; Group 2, HF diet plus Methanolic extract of *S. indicus* (100 mg/kg body weight/day); Group 3, HF diet plus methanolic extract of *S. indicus* (300 mg/kg body weight/day). Mice were fed pelleted HF diet, and test agent was administered by oral gavage in the morning once daily. New batches of dosing solution was prepared every week and stored as aliquots at 4 ^o^C. Food was replaced with fresh food at the intervals of every 3 days. Body weights were monitored every week, and feeding continued for 8 weeks. At the end of 8 weeks of treatment, mice were bled retro-orbitally under isoflurane anesthesia, and plasma analyzed for triglycerides, cholesterol, LDL-C, HDL-C, glucose, and cytokine level. Mice were sacrificed by carbon dioxide asphyxiation. Aorta were removed, and processed for Oil Red O staining as described [34, 35]. Aortae from 4 mice in each group were isolated without formalin fixing to prepare total RNA [30].

### Statistical analysis

Mean values of treated groups were compared to those of the vehicle treated group. Statistical significance was determined by ANOVA. Statistical comparisons across treatment groups were done. All results were presented as mean ± SD. A p value of <0.05 was considered significant.

## Results

### *S. indicus* extract decreases TNF-α stimulated pro-inflammatory cytokines in human peripheral blood monocytic cells

**Fig. 1:**
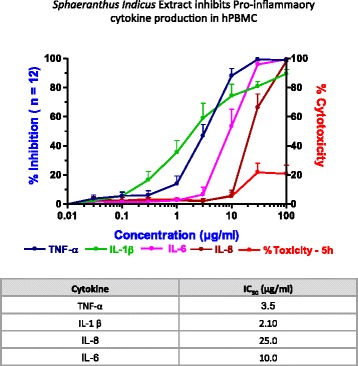
*Sphaeranthus Indicus* extract inhibits lipopolysaccharide-induced TNF-α production in human peripheral monocytic cells. Freshly isolated human peripheral blood monocytes were pretreated with *S. indicus* extract ranging in concentration from 0.01 to 100 μg/ml for 30 min followed by treatment with LPS and incubation for 6 h at 37 °C, 5 % CO_2_ followed with collection of supernatants and measurements for TNF-α, IL-6, IL-8, and IL-1β by ELISA Assay. The 50 % inhibitory concentration (IC_50_) values were calculated by a nonlinear regression method using GraphPad software

Screening of natural products for their anti-inflammatory activities were performed in monocytes isolated from human peripheral blood. To induce cytokine production, PBMC was stimulated with LPS and inhibition of cytokine secretion was measured. As shown in Fig. 1, *S. indicus* extract inhibited pro-inflammatory cytokines, TNF-α, IL1-β, IL-6, and IL-8. The concentration needed to achieve maximal inhibition for each cytokines was found to be different. Whereas significant inhibition in the secretion of IL1-β was seen at as low as 1 μg/ml concentration, it required 3 and 10 μg/ml *S. indicus* extract to have similar inhibition for TNF-α and IL-6 production. While 30 μg/ml *S. indicus* extract resulted in >80 % inhibition in TNF-α, IL1-β and IL-6 production, inhibition of IL-8 secretion was ~70 %. The IC_50_ for TNF-α, IL1-β, IL-6, and IL-8 were 3.5, 2.1, 10, and 25 μg/ml, respectively. The maximal efficacious concentration showed no toxicity in this study.

### *S. indicus* extract and 7-HF decrease the expression of VCAM-1, ICAM-1 and E-selectin by TNF-α stimulated HUVECs

**Fig. 2:**
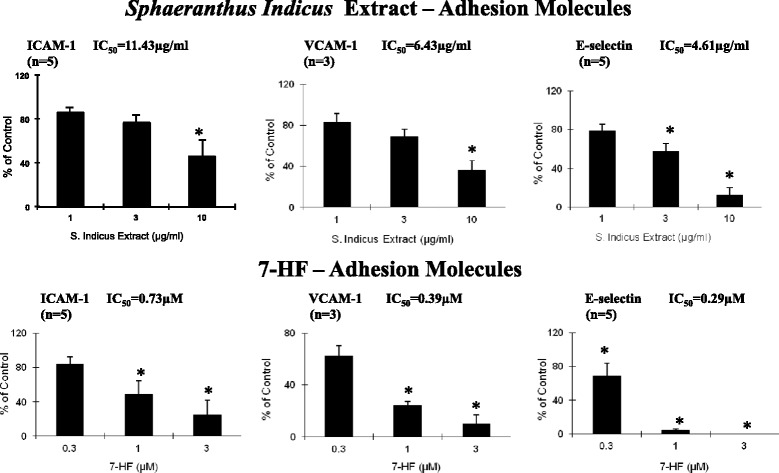
*Sphaeranthus Indicus* extract inhibits TNF-α-induced production of adhesion molecules human umbilical vein endothelial cells. Confluent HUVECs in 96-well fibronectin–coated plates were pretreated with various concentrations of *S. indicus* or 7-HF for 30 min before being stimulated with 1 ng/mL TNF-α for the indicated time. The expressions of ICAM-1 and E-selectin were evaluated after TNF-α stimulation for 4 h, and expression of VCAM-1 was evaluated after 6 h of stimulation. The 50 % inhibitory concentration (IC_50_) values were calculated by a nonlinear regression method using GraphPad software

As cell adhesion molecules play an important role during inflammation, we analysed the effect of different concentrations of *S. indicus* extract and 7-HF on TNF-α-induced cell surface expression of these molecules. In accordance with previous studies, ICAM-1 and E-Selectin were expressed at low levels in unstimulated HUVECs, but their expression was increased after TNF-α stimulation (data not shown). High-dosage (10 μg/ml) but not low-dosage (3 μg/ml) *S. indicus* extract significantly inhibited TNF-α induced ICAM-1 (54 ± 14 %), VCAM-1 (64 ± 9 %) and E-selectin (88 ± 8 %), respectively (Fig. 2). The IC_50_ values of *S. indicus* extract to ICAM-1, VCAM-1 and E-selectin expressio were 11.43, 6.43, and 4.61 μg/ml, respectively. 7-HF, at a concentration of 1 and 3 μM also significantly reduced the expression of ICAM-1 (52 ± 16 % and 75 ± 17 %), VCAM-1 (76 ± 3 % and 90 ± 7 %) and E-selectin (96 ± 2 % and 100 ± 0 %) respectively. The IC_50_ values of 7-HF to ICAM-1, VCAM-1 and E-selectin were 0.73, 0.39, and 0.29 μM, respectively. Taken together, these findings indicate that *S. indicus* extract as well as its active ingredient, 7-HF specifically inhibit cytokine-induced expression of adhesion molecules in a dose-dependent manner.

The cell cytotoxicity was assessed by MTS assay. Treatment of HUVECs with 1 ng/mL of TNF-α did not result in cytotoxicity (data not shown). When incubated with 1, 3 and 10 μg/mL of *S. indicus* extract, cell viability was 89 ± 3 %, 89 ± 3 % and 100 ± 0 % respectively. Treatment with 0.3, 1 and 3 μM did not affect cell viability (89 ± 3 %, 89 ± 3 % and 100 ± 0 % respectively).

### Effect of *S. indicus* extract on inhibition of TNF-α and IL-1β production in mice

**Fig. 3:**
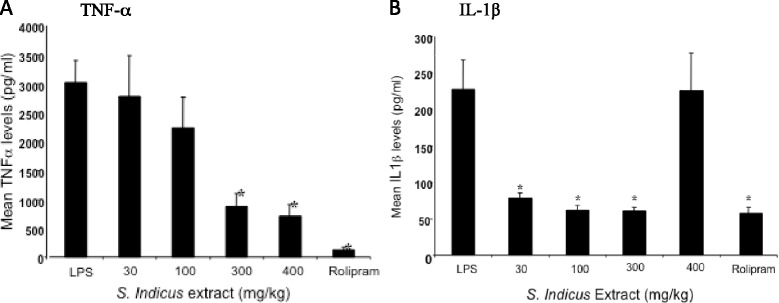
**a** *Sphaeranthus Indicus* extract inhibits lipopolysaccharide-induced TNF-α production in mice. Groups of 10 mice were treated orally with *Sphaeranthus Indicus* methanolic extract, 60 min prior to lipopolysaccharide injection (1 mg/kg, i.p.). Levels of TNF-α in the plasma were measured 1.5 h after lipopolysaccharide injection. Results are expressed as the mean ± S.E.M. *P < 0.001 significantly different from LPS control (students *t* test). **b** *Sphaeranthus indicus* extract inhibits lipopolysaccharide-induced IL-1β production in mice. Groups of 10 mice were treated orally with *S. indicus* 60 min prior to lipopolysaccharide injection (2 mg/kg, i.p.). Amounts of IL-1β in the plasma were measured 4 h after lipopolysaccharide injection. Results are expressed as the mean ± S.E.M. *P < 0.001 significantly different from LPS control (students *t* test)

Groups of 10 mice were treated orally with *Sphaeranthus Indicus* methanolic extract, 60 min prior to lipopolysaccharide injection (1 mg/kg, i.p.). Levels of TNF-α in the plasma were measured 1.5 h after lipopolysaccharide injection, and IL-1β were done 4 h after LPS treatment. The results shown in Fig. 3a suggest a dose-dependent inhibition of TNF-α production with 300 mg/kg dose of *S. indicus* extract showing maximal inhibition (~80 %). Rolipram, used as a reference agent, showed robust inhibition of LPS-induced TNF-α production. The effect of *S. indicus* extract on IL-1β was found to be even more robust (Fig. 3b), showing >60 % inhibition at 30 mg/ml concentration, which was as effective as rolipram. Heat inactivated and denatured extract (400 mg/kg dose) did not show any efficacy.

### Effect of *S. indicus* extract and 7-HF on NF-κB activation by TNF-α

**Fig. 4:**
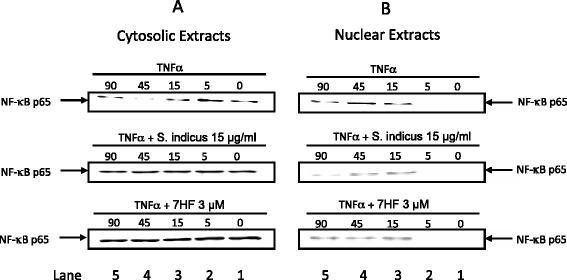
Effect of *S. indicus* extract and 7 HF on the nuclear translocation of NF-kB p65 in TNF-α-stimulated HUVECs. Briefly, confluent HUVEC monolayers were pretreated with 15 μg/mL *S. indicus* extract or 3 μM 7 HF for 1 h followed by stimulation with 0.1nM TNF-α for the time points. The cytosolic (Panel **a**) as well as nuclear (Panel **b**) extracts were prepared (as described in materials and methods) and analyzed by Western blot analysis with a primary antibody against human NF-kB p65 (3 independent experiments). Upper row shows treatment with TNF-α, middle rows shows treatment with TNF-α + S. indicus extract, and lower row shows TNF-α + 7-HF

The TNF-α induced expression of adhesion molecules ICAM-1, VCAM-1 and E-selectin requires the transcription factor NF-κB [36, 37]. NF-κB is also involved in the inflammatory diseases like arthritis [38] and psoriasis [39]. Therefore, we studied the effect of *S. indicus* extract and 7-HF on the nuclear translocation of the NF-κB p65. We carried out Western blot analysis to determine the levels of NF-κB p65 in the cytoplasmic and nuclear extracts from HUVEC treated with TNF-α in the presence or absence of *S. indicus* extract and 7-HF. As shown in Fig. 4a & b, TNF-α induced nuclear translocation of NF-κB p65 occurs within 15 min (Lane 4). In contrast, *S. indicus* extract and 7-HF, at a concentration of 15 μg/ml and 3 μM respectively, decreased the amount of nuclear NF-κB, with a concurrent increase in the amount of cytoplasmic NF-κB (Fig. 4a). Thus, *S. indicus* extract and 7-HF completely abolished nuclear translocation of NF-κB p65. Although equal amount of protein was taken for Western blotting, which was determined by a linear curve obtained from amount of protein loaded and the respective protein band intensity, these results should be interpreted cautiously.

### Effect of *S. indicus* extract and 7-HF on TNF-α-induced phosphorylation and degradation of IκBα

**Fig. 5:**
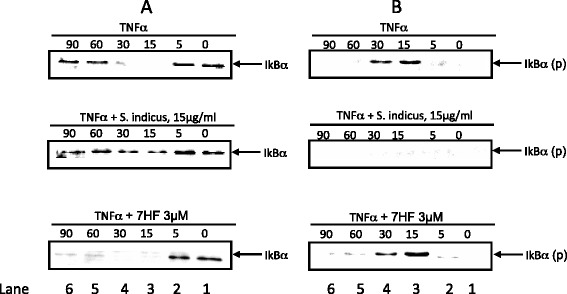
Effect of *S. indicus* extract and 7 HF on TNF-α-induced phosphorylation and degradation of IkB-α. Briefly confluent HUVEC monolayers were pretreated with 15 μg/mL *S. indicus* extract or 3 μM 7 HF for 1 h followed by stimulation with 0.1 nM TNF-α for the time points. The cytosolic extracts were prepared (as described in materials and methods) and then analyzed by Western blot analysis with a primary antibody against human IkB-α (**a**) phosphorylated IkB-α Κ (**b**) (3 independent experiments)

TNF-α-induced regulation of NF-κB involves the phosphorylation, ubiquitination and degradation of the cytoplasmic inhibitor, IκB-α [40]. To determine whether *S. indicus* extract and 7-HF inhibits NF-κB activation due to an effect on the phosphorylation and/or degradation of IκBα, the cytoplasmic IκBα protein levels were examined by Western blot analysis. The results show that a 37-kDa protein was detected in the cytoplasmic extract of untreated cells at time 0 min (Fig. 5a, lane 1) whereas TNF-α treatment caused a loss of IκB-α after 15 min (Fig. 5a, lane 3). Thereafter, IκB-α was reactivated 60 min onwards (Fig. 5a, lane 5 and 6), possibly by NF-κB, as NF-κB is known to bind and activate the IκB-α promoter. The pretreatment of cells with *S. indicus* extract at 15 μg/ml completely abolished TNF-α-induced degradation of IκB-α (Fig. 5a, lane 3 and 4), but 7-HF did not show similar results (Fig. 5a, lane 3 and 4). In fact, IκB-α was not resynthesized even after 60 min in presence of 7-HF (Fig. 5a, lane 5 and 6). This is not surprising since inhibition of NF-κB activity would also inhibit the regeneration of IκB-α mRNA.

To ascertain whether *S. indicus* extract inhibits IκB-α degradation by blocking its phosphorylation, the cyoplasmic extracts were examined by western blot analysis using antibodies that detect only the serine-phosphorylated form of IκB-α. The results show that the serine phosphorylated form of IκBα appears at 15 and 30 min (Fig. 5b, lanes 3 and 4), which completely disappeared when cells were pretreated with *S. indicus* extract (Fig. 5b, lanes 3 and 4), indicating that it completely abolished the TNF-α induced phosphorylation of IκB-α. However, the cells pretreated with 7-HF did not show similar results. While the differences in the untreated and treated groups are evident when equal amounts of protein was loaded to each lane, it should be noted that internal control was not included. Nevertheless, the results do indicate inhibition of IκBα phosphorylation.

Taken together, the cell ELISA and Western blot analysis, suggest that *S. indicus* extract inhibits TNF-α-induced IκBα phosphorylation and degradation, which causes a decrease in NF-κB resulting in decreased surface expression of adhesion molecules. In contrast, 7-HF, one of its bioactive principles, without affecting the phosphorylation and degradation of IκB-α, inhibits the activation of NF-κB thereby reducing the surface expression of adhesion molecules.

### Effect of *S. sphaericus* extract on plasma lipids and atherosclerosis in hamsters

**Fig. 6:**
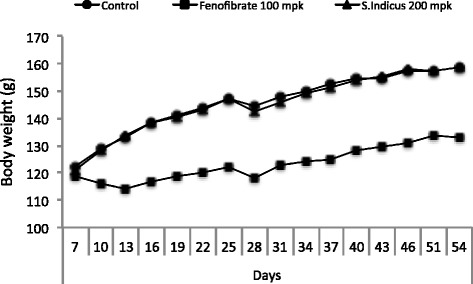
Effect of *S. indicus* on body weight in diet-induced hyperlipidemic hamsters. Body weight measurements were carried out 18 times at the interval of 3 days during the course of 60 days study

**Fig. 7:**
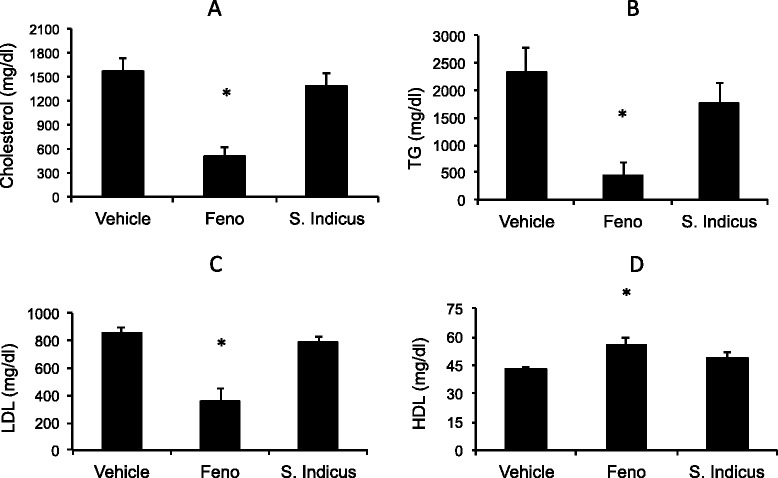
Effect of *S. indicus* extract on circulating lipids in diet-induced hyperlipidemic hamsters. Hamsters were fed on hyperlipidemic diet for 8 weeks and concurrently treated with *S. indicus* preparation. At the end of 8 weeks of treatment blood were withdrawn for lipid analyses. Panel **a**, Cholesterol; Panel **b**, Triglycerides; Panel **c**, LDL; Panel **d**, HDL

**Fig. 8:**
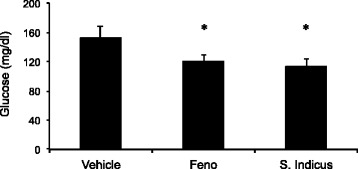
Effect of *S. indicus* extract on plasma glucose in diet-induced hyperlipidemic hamsters

Feeding high fat high cholesterol diet caused gradual increase in body weight (Fig. 6), showing a 30 % increase after 59 days. The treatment group showed no difference in body weight gain on high fat high cholesterol diet during the same period, suggesting that there was no adverse effect of the *S. indicus* extract treatment up to 200 mg/kg body weight/day in hyperlipidemic hamsters. The total cholesterol and triglycerides rose to 1700 mg/dl and 2200 mg/dl, respectively (Fig. 7). The hamster model of atherosclerosis is hyperlipidemia driven [31, 33]. The total cholesterol and LDL-C remained unchanged in the *S. indicus* extract treated group, however, there was a small change in triglyceride level. Fenofibrate, known to lower lipids in the hyperlipidemic hamsters [31, 33], showed similar hypolipidemic effect with massive reductions in total cholesterol, triglycerides and LDL-C (Fig. 7a-d). Since insulin resistance and diabetes may also influence arterial lipid deposition [41], we measured plasma levels of glucose shown in Fig. 8. There was a modest decrease in the glucose levels in *S. indicus* extract treated as well as fenofibrate treated groups.

**Fig. 9:**
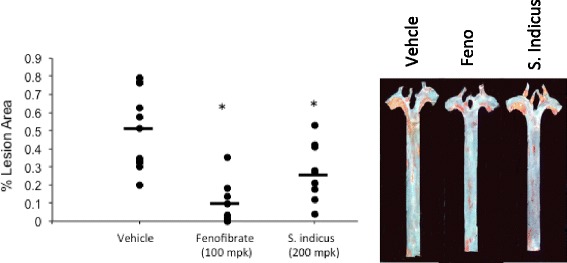
Effect of *S. indicus* extract on aortic lipid deposition in diet-induced hyperlipidemic hamsters. Animals were fed high fat high cholesterol diet and concomitantly dosed by oral gavage At the end of 10 weeks of treatment, hamsters were euthanized under CO_2_ and blood withdrawn by cardiac puncture and processed plasma lipid and glucose analysis. Their thorax was opened and vasculature perfused first with heparinized saline then with 10 % formalin. The aorta attached to heart and containing aortic arch, thoracic and abdominal aorta to the femoral artery bifurcation were removed and placed in 10 % formalin for en face staining and lesion quantitation. Aortic lipid contents were quantitated by en face staining with Oil Red O and atherosclerotic lesion area coverage determined by image analysis

In the control vehicle treated hamsters, 0.5 % of the aorta was found to have lesions (Fig. 9). Fenofibrate, included as a reference agent, reduced lesion area by more than 80 %. This reduction in lesion area was associated with massive reductions in proatherogenic lipoproteins. Treatment with *S. indicus* extract reduced aortic lesion by more than 40 % without changes in the proatherogenic lipoproteins.

### Effect of *S. sphaericus* extract on plasma lipids and atherosclerosis in LDLr^-/-^ mice

**Fig. 10:**
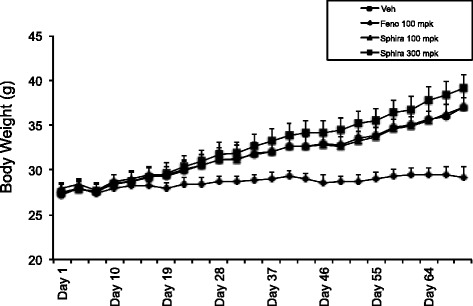
Effect of *S. indicus* on body weight in diet-induced hyperlipidemic LDL receptor-deficient mice. Body weight measurements were carried out at the interval of 3 days during the course of study

**Fig. 11:**
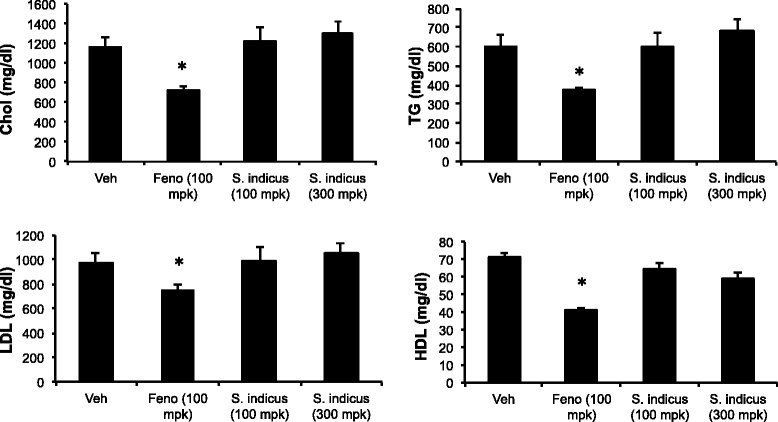
Effect of *S. indicus* extract on circulating lipids in diet-induced hyperlipidemic LDL receptor-deficient mice. Mice were fed Western Diet for 4 weeks followed by treatment with *S. indicus* preparation by oral gavage for 8 weeks. Mice were fed WD during treatment period. At the end of 8 weeks of treatment, mice were bled retro-orbitally under isoflurane anesthesia, and plasma analyzed for triglycerides, cholesterol, LDL-C, and HDL-C

**Fig. 12:**
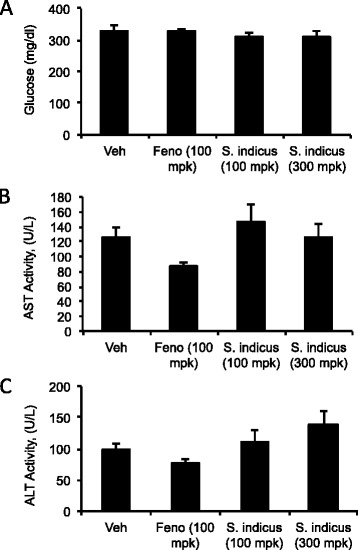
Effect of *S. indicus* extract on plasma glucose and markers of liver toxicity in diet-induced hyperlipidemic LDL receptor-deficient mice. Panel **a**, Glucose; Panel **b**, AST; Panel **c**, ALT

Anti-atherosclerotic and anti-hyperlipidemic activities of *S. indicus* extract were also evaluated in a mouse model of atherosclerosis at two concentrations, 100 and 300 mg/kg/d. While there was no change in body weight at 100 mg/kg/d dose (Fig. 10), a non-significant increase in body weight was noticed at 300 mg/kg/d dose, suggesting that this high dose was safe in animal model of atherosclerosis. As expected [30], fenofibrate prevented body weight gain. Feeding high fat high cholesterol diet raised total cholesterol and triglycerides ~1200 and ~600 mg/dl, respectively (Fig. 11). While fenofibrate decreased proatherogenic lipoproteins and triglycerides, *S. indicus* preparation did not change circulating lipoproteins and triglycerides (Fig. 11). Since a high dose of *S. indicus* preparation was used in this study, we also measured some of the parameters indicative of safety. As shown in Fig. 12, no significant changes were observed in AST and ALT levels.

**Fig. 13:**
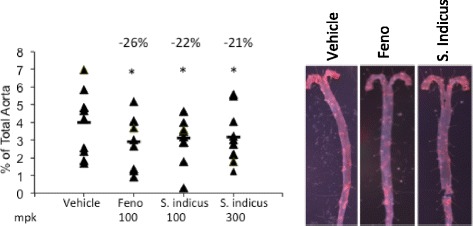
Effect of *S. indicus* extract on aortic lipid deposition in diet-induced hyperlipidemic LDL receptor-deficient mice. Mice were fed Western Diet for 4 weeks followed by treatment with *S. indicus* preparation by oral gavage for 8 weeks. Mice were fed WD during treatment period. At the end of 8 weeks of treatment, mice were bled retro-orbitally under isoflurane anesthesia, sacrificed by carbon dioxide asphyxiation, and aorta removed and processed for Oil Red O staining

High fat and cholesterol feeding to LDLr^-/-^ mice caused almost 4 % coverage of aorta with lipids as measured by neutral lipid staining. Treatment with fenofibrate showed a reduction of 26 % in lipid staining. *S. indicus* preparation reduced lesion area by 22 % at 100 mg/kg/day dose. Increasing the dose of *S. indicus* preparation did not show any advantage either in terms of overall lipid profile or the extent of reductions in lesion area (Fig. 13).

### Effect of *S. sphaericus* extract on cytokines in LDLr^-/-^ mice

**Fig. 14:**
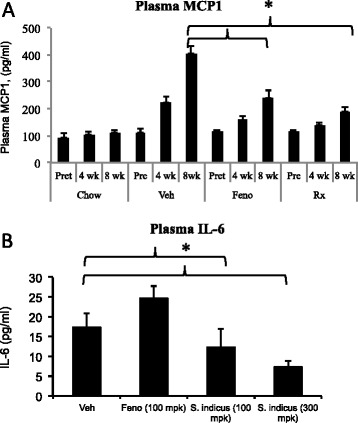
Effect of *S. indicus* extract on plasma MCP1 and IL-6 in diet-induced hyperlipidemic LDL receptor-deficient mice. MCP1 and IL-6 measurements were made in the blood samples collected at the time of terminal bleeding prior to removal of the aorta. Panel **a**, Plasma MCP1; and Panel **b**, Plasma IL-6

**Fig. 15:**
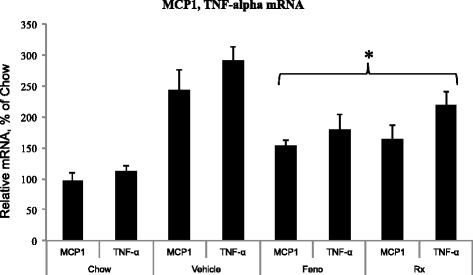
Effect of *S. indicus* extract on aortic MCP1 and TNF-α mRNA in diet-induced hyperlipidemic LDL receptor-deficient mice. Aortae from 3 animals from each group were not subjected to OilRed O staining. These aortae were used to prepare total RNA for the quantitation of MCP1 and TNF-α mRNA

To evaluate anti-inflammatory efficacy in atherosclerosis model, we measured circulating pro-inflammatory cytokines and adhesion molecules to ascertain its role in anti-atherosclerotic properties. Since IL-6 is a pro-inflammatory cytokine [42] and MCP-1, a cell adhesion molecule and a proatherogenic marker [43], we measured these two proteins in the serum. As shown in Fig. 14, there was 4-fold increase in the levels of MCP-1 as a result of high fat and cholesterol feeding, which decreased more than 2-fold following treatment with 100 mg/kg/day of *S. indicus* preparation. High fat high cholesterol diet caused only modest increases in IL-6 levels, however, treatment with *S. indicus* preparation caused significant reduction (~50 %) at 100 mg/kg/day dose and 75 % reduction at 300 mg/kg/day dose. To assess if the changes in MCP-1 occurred as a result of transcriptional activation, RNA from aorta was prepared and MCP-1 mRNA quantitated. There was a 2.5-fold increase in the aortic MCP-1 mRNA in the vehicle treated group when compared to MCP-1 level at the start of the high fat feeding, which decreased by 40 % following treatment with *S. indicus* preparation (Fig. 15). Fenofibrate treatment also showed similar reductions in MCP-1. Since the levels of TNF-α could not be reliably measured in the plasma because of low levels, we attempted to measure TNF-α mRNA in the RNA prepared from aorta. The results of TNF-α measurements shown in Fig. 15 suggest that high fat feeding does increase TNF-α mRNA in the aorta by ~3-fold. Treatment of high fat-fed LDLr^-/-^ mice with *S. indicus* preparation caused a ~30 % reduction in TNF-α mRNA. Both IL-6 and IL1-β did not show significant changes (data not shown).

## Discussion

In the course of screening natural products to find novel anti-inflammatory drugs as TNF-α inhibitors we found that *S. indicus* extract and 7-HF, one of its active constituents, characterized as a sesquiterpene lactone, suppresses the cytokine production in a concentration-dependent manner from LPS-stimulated human peripheral blood mononuclear cells as well as from synovial cells obtained from rheumatoid arthritis patients undergoing knee replacement surgery. Most importantly, *S. indicus* extract was also found to arrest disease progression in an in vivo mouse model for rheumatoid arthritis [29]. Since atherosclerosis is an inflammatory disease [44], we sought to investigate if anti-inflammatory activities of *S. indicus* inhibited atherosclerosis progression. We also looked into the mechanism of anti-inflammatory properties of *S. indicus.*

It has been previously documented that sesquiterpene lactones or sesquiterpene lactone-containing plant extracts exert potent anti-inflammatory effect, at least in part, through inhibition of transcription factor NF-κB [45], causing a decrease in adhesion molecule expression and subsequent leukocyte adhesion. Here, we show that natural product, *S. indicus* extract along with one of its active constituent, 7-HF mediate anti-inflammatory effects by effectively modulating the expression of cell adhesion molecules via suppression of NF-κB/IκBα signaling in TNF-α activated HUVECs.

Clearly, *S. indicus* extract (at the dose of 10 μg/ml) significantly inhibited the expression of the ICAM-1 (54 % inhibition), VCAM-1 (64 % inhibition), and E-selectin (88 % inhibition) in TNF-α activated HUVECs (Fig. 2). Likewise, 7-HF, a sesquiterpene lactone, (at the dose of 3 μM) dramatically inhibited ICAM-1 (75 % inhibition), VCAM-1 (90 % inhibition), and E-selectin (100 % inhibition) in TNF-α treated HUVECs (Fig. 2). Similar effects with compounds such as 1,4-Dihyroxyxanthone (65 μg/mL) [46], diclofenac (750 μM) [47], pyrrolidine dithiocarbamate (1 mM) [48] and N-acetyl cysteine (100 μM) [49] on cytokine-induced expression of cell adhesion molecules have been demonstrated by previous investigators, albeit at higher concentrations. Interestingly, *S. indicus* extract and one of its bioactive components, 7-HF, were found to be effective at comparatively lower concentrations and therefore, may be used in controlling various pathological conditions associated with upregulation of endothelial leukocyte adhesion molecules such as atherosclerosis, bacterial sepsis, inflammation and tumor metastasis.

To further delineate the mechanism by which *S. indicus* extract and 7-HF may be acting, we studied its effect on NF-κB activation. Using Western blot analysis, we show that *S. indicus* extract and 7-HF both appear to block TNF-α induced p65 translocation from the cytoplasm to the nucleus. In this respect, 7-HF possibly behaves differently from other known sesquiterpene lactones [45] such as helenalin [50], parthenolide [51] and isodeoxyelephantopin [52], which exert their anti-inflammatory effect by either targeting the IκB kinase complex or inhibiting the DNA binding of p65 homodimer. The mode of action of 7-HF appears to be similar to compounds like caffeic acid phenethyl ester (CAPE) [53] and DHMEQ [54], which block NFκB by inhibiting its binding to promoter DNA without affecting the degradation of IκB-α. Because NF-κB is responsible for the induced expression of ICAM-1, VCAM-1 and E-selectin [55], these results suggest that both these agents inhibit NF-κB dependent transcription of cell adhesion molecule genes. However, it is quite possible that *S. indicus* extract and 7 HF may also inhibit other transcription factors.

In addition, we also showed that pretreatment of cells with proteasome inhibitor ALLN prevents the destruction of IκB-α and results in accumulation of phosphorylated IκB-α. On the other hand, treatment of cells with *S. indicus* extract did not result in the accumulation of phosphorylated IκB-α in the cytoplasm. This led us to conclude that *S. indicus* extract possibly prevents NF-κB by preventing phosphorylation of IκB-α rather than proteolysis per se [40]. It is well known that the pathway leading to activation of NF-κB is common for these activators, implying that *S. indicus* extract acts at a molecular step common to both the LPS- and TNF pathways, which could be at the level of IKK or upstream of IKK. The effect of *S. indicus* extract on the cascade upstream of IKK remains to be clarified, although it is quite possible that *S. indicus* extract may suppress different steps in the NF-κB activation pathway.

The standardized *S. indicus* extract (10 μg/ml) contains approximately 0.6 μg/ml or 3 μM of 7-HF, and since we observed similar effects of *S. indicus* extract at 10 μg/ml and 7-HF at 3 μM (data not shown), we postulated that the effects on the expression of cell adhesion molecules and NF-κB activation seen with the crude extract of *S. indicus* extract may be related to its content of 7-HF. However, there are as yet uncharacterized active components besides 7-HF, which may be responsible for the additional potency of *S. indicus* extract that is reflected in the inhibition of IκB-α phosphorylation and degradation.

Thus, *S. indicus* extract and 7-HF a) significantly reduced ICAM-1, VCAM-1 and E-selectin expression; b) markedly inhibits the translocation of p65 from cytoplasm to the nucleus; and c) inhibits IκB-α phosphorylation and degradation. These activities of *S. indicus* extract and 7-HF suggest its potential to treat a wide variety of NF-κB-linked pro-inflammatory diseases such as rheumatoid arthritis, atherosclerosis and inflammatory bowel disease.

We, therefore, evaluated anti-atherosclerotic activities of *S. indicus* preparation in two widely studied animal models of atherosclerosis. Based on our in vitro results, we chose a dose equivalent to 120 mg/kg/day of active ingredient, 7-HF to examine the potential of *S. indicus* preparation to treat atherosclerosis. Both hamster and the LDLr^-/-^ mice showed efficacy in terms of slowing down atherosclerosis progression without modulating circulating proatherogenic lipoproteins, suggesting that the reductions in lesion area was not due to proatherogenic lipoproteins, but other mechanism(s). This effect was very different than those observed with fenofibrate, where reductions in lesion area were associated with reductions in proatherogenic lipoproteins in hamsters [33] as well as in LDLr^-/-^ mice [30]. Reductions in IL-6, TNF-α, and MCP-1 do suggest that *S. indicus* preparation decreased aortic lipid deposition by reducing circulating pro-inflammatory cytokines. These data are consistent with in vitro cell-based findings that showed NF-kB-mediated down-regulation of pro-inflammatory cytokines and adhesion molecules. Natural products that inhibits NF-kB pathway lower cell adhesion molecules and pro-inflammatory cytokines, leading to reduced atherosclerotic burden [56–58] without changes in circulating lipoprotein levels.

We hypothesized that anti-inflammaory agents showing efficacy in inflammatory diseases like psoriasis, colitis and rheumatoid arthritis will inhibit atherosclerosis progression. Indeed, individuals with psoriasis [18, 20, 59–62], inflammatory bowel disease [63], and rheumatoid arthritis [22, 25, 64–67] have greater risks of developing coronary artery disease. These findings together with chronic inflammation in atherosclerosis [5, 68] and end stage kidney disease in diabetics [69] have led to increasing efforts to discover and develop anti-inflammatory agents to treat coronary artery disease [70]. NFkB inhibitors are among anti-inflammatory agents showing anti-atherosclerotic activities [57, 58, 71]. High-density lipoproteins shown to regress atherosclerosis is believed to have an aniinflammatory component [72], in addition to promoting reverse cholesterol transport [73]. The anti-atherosclerotic activities of many of the natural products like alicin [74], catechins [75], abscisic acid [76], marine-derived wax esters [77], dried plums [78], and a number of plant-derived flavonoids [79], are attributed to their anti-inflammatory activities. The anti-atherosclerosis efficacy of *S. indicus* preparation in the present study is not surprising, as a number of anti-inflammatory agents possess anti-atherosclerosis activities via NF-kB mediated pathways [57, 58, 71]. Sesquiterpene lactones have been shown in apoE^-/-^ mice and guinea pigs to have anti-atherosclerosis efficacy via their anti-inflammatory activities [80, 81]. Here we showed that *S. indicus* preparation with major ingredient 7-HF, a sesquiterpene lactone, inhibited atherosclerosis progression in two diet-induced atherosclerosis models, LDLr knockout and hamsters.

In summary, we showed that the main component of *S. indicus* preparation, 7-HF, is an anti-inflammatory agent that works through NF-kB-mediated pathway, and inhibits atherosclerosis progression. These findings offer promise for further clinical benefits to patients with inflammatory and cardiovascular diseases.
